# The Relationship between Interleukin-18 Polymorphisms and Allergic Disease: A Meta-Analysis

**DOI:** 10.1155/2014/290687

**Published:** 2014-06-05

**Authors:** Daye Cheng, Yiwen Hao, Wenling Zhou, Yiran Ma

**Affiliations:** Department of Transfusion, The First Hospital of China Medical University, North Nanjing Street, No. 155, Shenyang, Liaoning 110001, China

## Abstract

Recent studies have suggested that *IL-18* −607C/A and −137G/C polymorphisms may be associated with the risk of allergic disease; however, individually published results are inconclusive. Therefore, we performed a meta-analysis to clarify whether *IL-18* −607C/A and −137G/C polymorphisms were associated with the risk of allergic disease. A total of 21 studies including 5,331 cases and 9,658 controls were involved in this meta-analysis. In the overall analysis and the subgroup analysis according to ethnicity, we did not find significant association between *IL-18* −607C/A or −137G/C polymorphism and the risk of allergic disease (all *P* > 0.05). However, in a stratified analysis by type of allergic disease, our results indicated that *IL-18* −607C/A polymorphism was associated with a significantly decreased risk of allergic asthma in heterozygous comparison and *IL-18* −137G/C was associated with a significantly decreased risk of allergic dermatitis in recessive model and homozygous comparison. In the stratified analysis by source of control, *IL-18*−607C/A showed significantly reduced risk in population-based subgroup, and for *IL-18* −137G/C only significantly decreased risk was found in the hospital-based subgroup. Our meta-analysis suggests that *IL-18* −607C/A and −137G/C polymorphisms may be protective factors for the risk of allergic asthma and allergic dermatitis, respectively.

## 1. Introduction

People with allergic disorders such as allergic dermatitis (AD), allergic rhinitis (AR), allergic asthma (AA), and food allergy can experience acute signs and symptoms of disease within minutes of exposure to the associated allergens [1]. To date, allergic diseases are causes of tremendous morbidity, and the rise in allergic disease is fast becoming a major global health issue [2, 3]. However, the reasons and mechanisms are not well understood. Accumulating evidence demonstrated that allergic diseases are complex genetic diseases resulting from the effect of multiple genetic and interacting environmental factors on their pathophysiology. The nature of the individual genes that have been identified as susceptibility factors for allergic disease has been comprehensively reviewed elsewhere, and the list of these genetic factors is likely to expand considerably in the future with the recent advent of genome-wide association approaches [4–6].

IL-18, formerly called interferon- (IFN-) *γ*-inducing factor, is a novel cytokine belonging to the IL-1 family and is produced by a wide range of immune cells, such as monocytes, activated macrophages, and Kupffer cells [7–9]. IL-18 has become recognized as an important regulator of innate and acquired immune responses and is expressed at sites of chronic inflammatory disease [10]. To date, accumulating evidence demonstrated that IL-18 synergizes with IL-12 to induce IFN-*γ* production and promote Th1 responses [11, 12]. However, IL-18 alone can also induce Th2 cytokine, IL-13, and promote Th2 responses [13, 14]. Indeed, abnormal imbalance of Th1 and Th2 functions contributes to the pathogeneses of allergic disorders [15–17]. IL-18 was also involved in allergic inflammatory reactions by indirectly inducing B cell isotype switching to IgE and by inducing the production of Th2 cytokines such as IL-4 and IL-13 [18]. In addition, IL-18 is generally considered to be involved in the Th1-mediated immune response and inhibits IgE synthesis, usually by acting synergistically with IL-12 [19]. Taken together, these studies show that IL-18 may be having both allergy-promoting and antiallergic functions. More recently, positive relationship has been found between IL-18 levels in the lesion or circulation and allergic diseases, such as AA, AR, and AD [20]. However, IL-18 was also raised in patients with other diseases in which dominant Th1 cells play a key role for immunity [21–24]. These findings confirm the evidence of an association between IL-18 gene and allergic disease but remain controversial.

*IL-18* gene is located on chromosome 11q22 and contains many functional polymorphisms in the promoter region. Many studies show that the variations in* IL-18* gene promoter are able to influence IL-18 production and activity, especially the* IL-18* gene −607C/A (rs1946518) and −137G/C (rs187238) polymorphisms [25]. Giedraitis et al. reported that* IL-18* −607C/A can alter a cAMP-responsive element binding site and result in a decrease of* IL-18* transcription [26]. The* IL-18* −137G/C polymorphism leads to a G/C amino acid substitution at position −137 in the promoter region of the* IL-18* gene, which could alter the* IL-18* promoter activity [27, 28]. Recent studies have suggested that* IL-18* polymorphisms may be associated with the risk of allergic disease; however, individually published results are inconclusive due to small sample sizes. Therefore, we performed a meta-analysis of all eligible studies to clarify whether these two polymorphisms of* IL-18* gene were associated with the risk of allergic disease, which may promote our understanding of the exact role of* IL-18* in the development of allergic disease.

## 2. Material and Methods

### 2.1. Search Strategy

A systematic search was performed in PubMed, Web of Science, Science Direct, and Chinese National Knowledge Infrastructure (CNKI) databases to identify all the studies on the association between* IL-18* −607C/A and/or −137G/C polymorphisms and allergic disease susceptibility (last search updated to November 2013). The search strategies were based on combinations of the following key words: “allergy or allergic disease or allergic disorder” and “*IL-18* or −607C/A or −137G/C or rs1946518 or rs187238” and “polymorphism or variant or mutation or genotype or SNP.” There was no limit on time period, sample size, population, or language for minimizing potential publication bias.

### 2.2. Selection Criteria

Studies consistent with the following criteria were included in the meta-analysis: (1) case-control studies focus on the associations between IL-18 promoter polymorphisms and the risk of allergic disease; (2) all patients meet the diagnostic criteria for allergic disease; (3) sufficient published data for estimating the odds ratio (OR) and their corresponding 95% confidence interval (95% CI). The exclusion criteria of the meta-analysis were (1) studies with duplicate data; (2) studies with incomplete data; and (3) reviews, abstracts, and meta-analysis. In the case of several articles from the same study group, the most complete and recent results were used.

### 2.3. Data Extraction

Two authors independently extracted the data from the included studies (Cheng and Hao). For each eligible study, the following information was extracted: first author's name, year of publication, country, ethnicity, type of allergic disease, numbers of cases and controls, source of controls (hospital-based controls or population-based controls), genotyping method, and Hardy-Weinberg equilibrium (HWE). In case of discrepancies, a consensus on each item was reached among the authors.

### 2.4. Statistical Analysis

The ORs with 95% CIs for genotypes were used to evaluate the strength of the association between* IL-18* −607C/A and −137G/C polymorphisms and the risk of allergic disease. The pooled ORs were calculated for allele model (mutation [M] allele versus wild [W] allele), dominant model (WM + MM versus WW), recessive model (MM versus WM + WW), homozygote comparison (MM versus WW), and heterozygote comparison (WM versus WW), respectively. The significance of the combined ORs was determined by a *Z*-test and two-sided *P* value <0.05 was considered significant.

Chi square-based *Q*-test and the *I* ^2^ statistic were performed to evaluate possible heterogeneity (*P* < 0.10 and *I* ^2^ > 50% indicated evidence of heterogeneity). A random-effects model or fixed-effects model was used to calculate pooled OR in the presence or absence of heterogeneity, respectively. Subgroup analyses were conducted according to the type of allergic disease, ethnicity, and source of control. Sensitivity analysis was carried out by deleting one single study each time to examine the influence of individual data set on the pooled ORs. Publication bias was evaluated with Begg's funnel plot [29] and Egger's regression method [30], and *P* < 0.05 was considered representative of statistically significant publication bias. Data analyses were performed using Stata 11.0 (StataCorp, College Station, TX) and Review Manager software 5.0 (Oxford, England).

## 3. Results

### 3.1. Characteristics of the Eligible Studies

**Figure 1:**
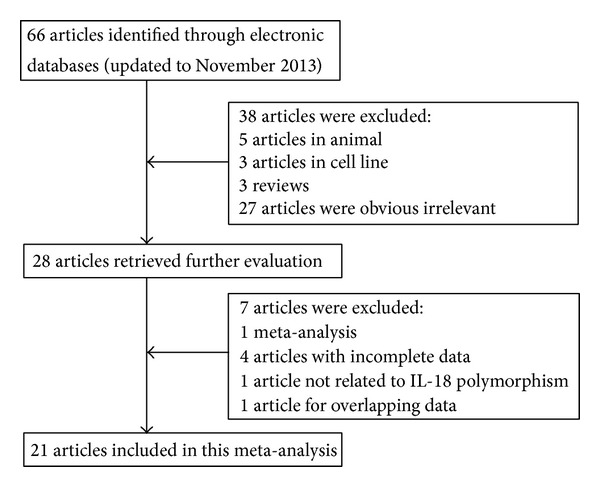
Flow diagram of studies included and excluded in the present meta-analysis.

As shown in Figure 1, a total of 66 potentially relevant articles were identified from PubMed, Web of Science, Science Direct, and CNKI databases using different combinations of key terms. After reading the titles and abstracts, we excluded 38 articles that assessed unrelated polymorphisms, were not case-control studies, were conducted in cell lines, and were performed in animal model. After reading the full texts of the remaining 28 articles regarding the association between* IL-18* polymorphisms and allergic disease, seven articles were excluded, 1 for meta-analysis related to IL-18 promoter polymorphism and asthma risk, 4 with incomplete data (no available genotype frequency), 1 not related to IL-18 polymorphism, and 1 for overlapping data. Finally, a total of 21 articles were identified for data extraction and assessment, including 5,331 cases and 9,658 controls. The studies identified and their main characteristics were summarized in Table 1. Of the 21 case-control studies included, an array of allergic diseases including AA [31–40], AD [41–45], AR [10, 46], drug allergy [47, 48], and other types [49, 50] was involved. Of all studies included, 12 studies were conducted in Asian populations [31, 33, 36, 37, 39, 40, 43–48], while 9 studies were performed in Caucasian populations [10, 32, 34, 35, 38, 41, 42, 49, 50]. There were 16 studies concerning* IL-18* −607C/A [10, 31, 32, 35–40, 44–50] and 19 studies concerning −137G/C [10, 31–37, 39–45, 47–49]. The genotype distributions among the controls of all studies were in agreement with HWE, except 2 studies for −607C/A [38, 46] and 1 study for −137G/C [33].

### 3.2. Quantitative Analysis

A summary of associations between* IL-18* −607C/A and −137G/C polymorphisms and allergic disease risk was shown in Table 2.

**Figure 2:**
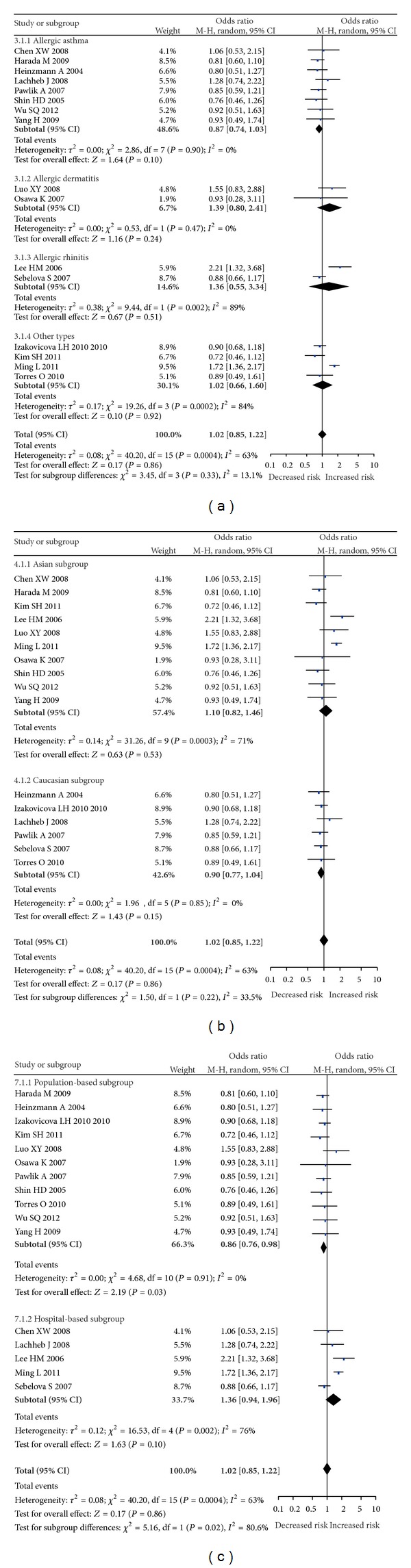
Forest plots for* IL-18* −607C/A polymorphism and allergic disease risk under dominant model. (a) Subgroup analysis by type of disease. (b) Subgroup analysis by ethnicity. (c) Subgroup analysis by source of control.

For* IL-18* −607C/A polymorphism, a total of 4,089 cases and 3,840 controls were included in the meta-analysis. There was significant between-study heterogeneity among all the genetic models (allele model: *I* ^2^ = 63%; dominant model: *I* ^2^ = 63%; recessive model: *I* ^2^ = 57%; homozygous comparison: *I* ^2^ = 71%; heterozygous comparison: *I* ^2^ = 62%). Therefore, random-effect model was applied to synthesize the data. In the overall analysis, we did not find significant association between* IL-18* −607C/A polymorphisms and the risk of allergic disease under all models (A allele versus C allele: OR = 1.03, 95% CI = 0.91–1.15, *P* = 0.67; AA + AC versus CC: OR = 1.02, 95% CI = 0.85–1.22, *P* = 0.86; AA versus AC + CC: OR = 1.07, 95% CI = 0.88–1.28, *P* = 0.51; AA versus CC: OR = 1.01, 95% CI = 0.77–1.33, *P* = 0.94; AC versus CC: OR = 1.00, 95% = 0.83–1.21, *P* = 0.98, Figure 2). In the subgroup analysis by the type of allergic disease, we only found significant association between* IL-18* −607C/A polymorphism and AA under heterozygous comparison (AC versus CC: OR = 0.82, 95% CI = 0.69–0.98, *P* = 0.03). In a stratified analysis by ethnicity, there was no significant association between* IL-18* −607C/A polymorphism and the risk of allergic disease (all *P* > 0.05 under all models) in Asian and Caucasian populations. In a stratified analysis by source of control,* IL-18* −607C/A showed significantly reduced risk in dominant model (OR = 0.86, 95% CI = 0.76–0.98, *P* = 0.03), homozygous comparison (OR = 0.80, 95% CI = 0.68–0.95, *P* = 0.009), and heterozygous comparison (OR = 0.85, 95% CI = 0.74–0.98, *P* = 0.03) in population-based subgroup but showed increased risk in allele model (OR = 1.27, 95% CI = 1.03–1.57, *P* = 0.03) and homozygous comparison (OR = 1.75, 95% CI = 1.14–2.68, *P* = 0.01) in hospital-based subgroup.

**Figure 3:**
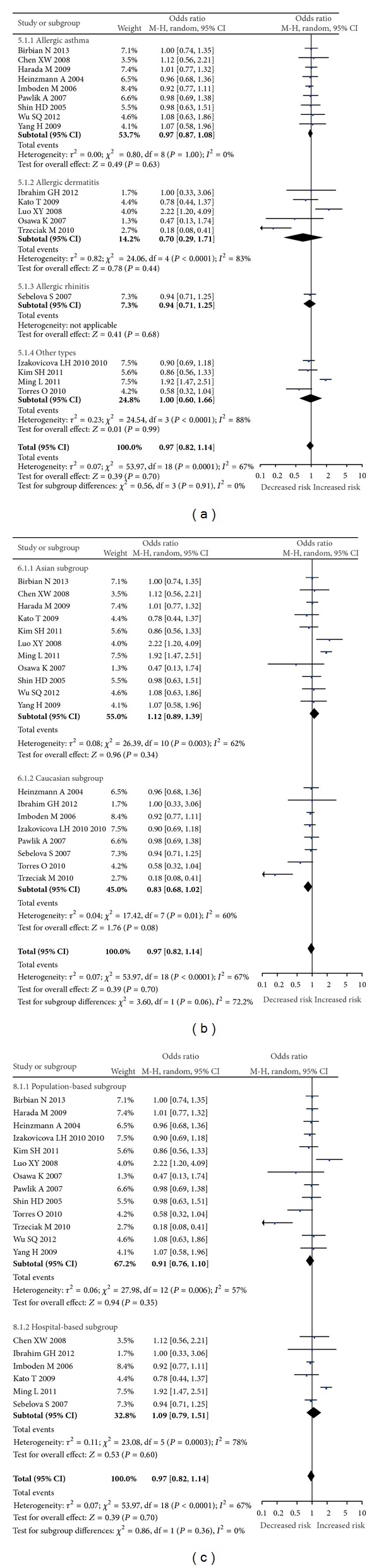
Forest plots for* IL-18* −137G/C polymorphism and allergic disease risk under dominant model. (a) Subgroup analysis by types of disease. (b) Subgroup analysis by ethnicity. (c) Subgroup analysis by source of control.

For* IL-18* −137G/C polymorphism, a total of 5,067 cases and 9,379 controls were included in the meta-analysis. We found significant between-study heterogeneity under allele model (*I* ^2^ = 73%), dominant model (*I* ^2^ = 67%), and heterozygous comparison (*I* ^2^ = 56%) and the random-effects model was used, whereas the fixed-effect model was used in recessive model (*I* ^2^ = 39%) and homozygous comparison (*I* ^2^ = 46%). In the overall analysis, we did not find significant association between* IL-18* −137G/C polymorphism and the risk of allergic disease under all models (C allele versus G allele: OR = 0.93, 95% CI = 0.80–1.07, *P* = 0.31; CC + CG versus GG: OR = 0.97, 95% CI = 0.82–1.14, *P* = 0.70; CC versus CG + GG: OR = 0.77, 95% CI = 0.58–1.02, *P* = 0.07; CC versus GG: OR = 0.76, 95% CI = 0.55–1.04, *P* = 0.08; CG versus GG: OR = 1.02, 95% CI = 0.88–1.18, *P* = 0.78, Figure 3). When the data were stratified by the type of allergic disease, a significant association between* IL-18* −137G/C polymorphism and AD was found under recessive model (CC versus CG + GG: OR = 0.30, 95% CI = 0.15–0.60, *P* < 0.001) and homozygous comparison (CC versus GG: OR = 0.26, 95% CI = 0.12–0.53, *P* < 0.001). In the subgroup analysis by ethnicity, the results suggested that* IL-18* −137G/C polymorphism was not associated with the risk of allergic disease (all *P* > 0.05 under all models) in Asian and Caucasian populations. In a stratified analysis by source of control, only significantly decreased risk was found in the hospital-based subgroup under recessive model (OR = 0.72, 95% CI = 0.54–0.96, *P* = 0.03) and homozygous comparison (OR = 0.72, 95% CI = 0.53–0.97, *P* = 0.03).

### 3.3. Sensitivity Analysis

In order to assess the reliability of our results, we performed a sensitivity analysis by sequentially excluding individual study. Statistically similar results were obtained after sequentially excluding each study, suggesting the stability of this meta-analysis.

### 3.4. Publication Bias

**Figure 4:**
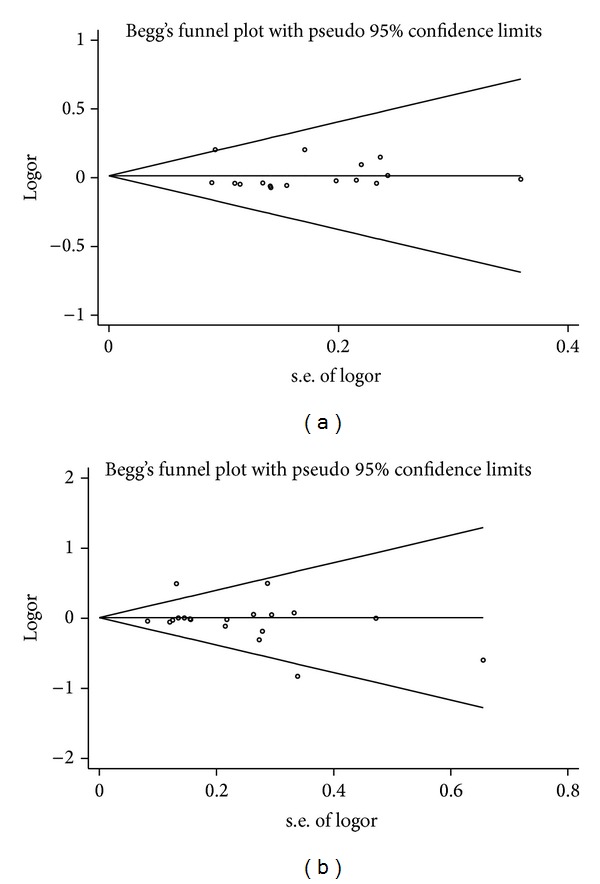
Publication bias represented by Begg's funnel plot for the association between* IL-18* polymorphisms and the risk of allergic disease under the dominant model. (a)* IL-18* −607C/A polymorphism; (b)* IL-18* −137G/C polymorphism.

Potential publication bias in this meta-analysis was estimated using Begg's funnel plot and Egger's test. The shape of funnel plot revealed the evidence of funnel plot symmetry for* IL-18* −607C/A and −137G/C polymorphisms (Figure 4), and Egger's test provided statistical evidence which identified the absence of publication bias (*IL-18* −607C/A, *P* = 0.741;* IL-18* −137G/C, *P* = 0.438).

## 4. Discussion

The enormous health importance of allergy disease has stimulated much work aimed at identifying susceptibility genes. Investigation into common genetic variation in the human genome has highlighted the contribution of genetics to etiology and pathogenesis of allergic diseases. However, findings are not always consistent. Considering a single study may lack statistical power to provide compelling evidence as a result of small sample size and clinical heterogeneities, we performed this meta-analysis by combining all eligible publications to derive an accurate assessment of the association between* IL-18* polymorphisms and the risk of allergic disease.

IL-18, belonging to the IL-1 superfamily, is a pleiotropic proinflammatory cytokine, which functions as a crucial regulator of IgE production through balancing the TH1-cell- and TH2-cell-mediated immune responses. A surfeit of IL-18 has been found in patients with allergic diseases, including AA, AD, and AR, in which a predominance of Th1 cells is significant [9, 20, 51]. Yoshimoto et al. suggested that IL-18 may be critical in regulation of IgE production* in vivo*, providing a potential therapeutic target for allergic disorders [52]. Therefore, the functions of IL-18 are very heterogeneous and complicated. In principle, IL-18 enhances the IL-12-driven Th1 immune responses, but it can also stimulate Th2 immune responses in the absence of IL-12 [53]. All of these findings suggest that IL-18 plays important roles in both antiallergic and allergy-promoting effects. Currently,* IL-18* genetic polymorphisms have been postulated to be implicated in the development of allergic disease. Large quantities of evidence support the hypothesis that* IL-18* genetic polymorphisms may cause the abnormal expression of IL-18, which is responsible for mediating the T-cell regulation, thereby leading to the pathological development of allergic disease.

In the present meta-analysis, including 5,331 cases and 9,658 controls,* IL-18* −607C/A and −137G/C polymorphisms were not found to be associated with the risk of allergic disease in the overall analysis. The conclusion of this meta-analysis is different from the previous meta-analysis [54]. As we knew, this meta-analysis is the latest one and has the largest sample size. So the result of our meta-analysis is more likely convincing.

Subgroup analyses according to ethnicity revealed similar results, suggesting that the environment they lived in did not play an obvious role in the association between* IL-18* polymorphisms and the risk of allergic disease. However, in a stratified analysis by types of allergic disease, our results indicated that* IL-18* −607C/A polymorphism was associated with a significantly decreased risk of AA in heterozygous comparison, and IL-18 −137G/C was associated with a significantly decreased risk of AD in recessive model and homozygous comparison. As mentioned above,* IL-18* gene promoter polymorphisms at positions −607C/A and −137G/C were associated with their transcription activity. Low promoter activity was observed for the −607A and −137C alleles, whereas higher promoter activity was observed for the −607C and −137G alleles in these positions [54]. Our meta-analysis showed that IL-18 −607C/A and −137G/C polymorphisms might be involved in the etiology of allergic disease, revealing a significant protective role or decreased risk towards AA and AD, respectively. Allergic disease is a multifactorial disease influenced by interactions of multiple susceptibility genes and environmental factors, and there will not be a single gene or single environmental factor that has a large effect on allergic disease susceptibility. However, our results should be interpreted with much caution. Only eight case-control studies for AA and five for AD were included in this meta-analysis, which might reduce statistical power to get a reliable result. In subgroup analysis, according to the source of control, significantly decreased risk was found in the population-based subgroup for* IL-18* −607C/A under three models (dominant model, homozygous comparison, and heterozygous comparison), but significant increased risk was found in hospital-based subgroup under two models (allele model and homozygous comparison). As for* IL-18* −137G/C, only significantly decreased risk was found in the hospital-based subgroup under recessive model and homozygous comparison. The reason may be that the hospital-based controls have a high risk of producing unreliable results because hospital-based controls may not always be strictly healthy individuals and hospital-based controls may have other diseases and also been given the corresponding drugs which exerted a confounding effect on the risk for allergic disease. Thus, we cannot completely exclude the possibility that a true genetic effect was overestimated, and the results of hospital-based studies should be explained with caution. Therefore, a proper and representative population-based high quality study is of great value in case-control studies.

There are several limitations in this meta-analysis that should be considered. First, there is a significant heterogeneity in studies, which may influence the interpretation of the results. Second, in subgroup analysis by ethnicity, the included studies were only Asians and Caucasians. More studies containing the full range of possible ethnic differences in genotype association studies are needed to avoid selection bias. Third, only published data was collected, leading to possible publication bias in this meta-analysis. Forth, it is well acknowledged that many other factors, such as gene-gene or gene-environment interactions, may affect the risk of allergic disease. Determining whether or not these factors influence the results of this meta-analysis would need further investigation.

## 5. Conclusion

In conclusion, our meta-analysis provided a more precise estimation based on larger sample size compared with the individual studies. Our pooled results demonstrated no significant association between* IL-18* polymorphisms and allergic disease. The subgroup analysis indicated that* IL-18* −607C/A and −137G/C polymorphisms may be a protective factor for the risk of AA and AD, respectively. In the future, more large-scale studies should be carried out to confirm or refute the relationship between IL-18 polymorphisms and the risk of allergic disease.

## Figures and Tables

**Table 1:** Characteristics of studies included in the present meta-analysis.

Studies	Year	Country	Ethnicity	Type	Source of controls	Sample size	SNP studied	Genotyping method	HWE
Harada et al. [31]	2009	Japan	Asian	AA	PB	453/719	−607C/A; −137G/C	Taqman	0.29; 0.48
Heinzmann et al. [32]	2004	German	Caucasian	AA	PB	230/269	−607C/A; −137G/C	Taqman	0.68; 0.17
Birbian et al. [33]	2013	Indian	Asian	AA	PB	410/414	−137G/C	ARMS-PCR	<0.001
Yang et al. [40]	2009	China	Asian	AA	PB	102/100	−607C/A −137G/C	PCR-SSP	0.08; 0.74
Imboden et al. [34]	2006	Swiss	Caucasian	AA	HB	530/5204	−137G/C	Taqman	0.37
Lachheb et al. [38]	2008	Tunisia	Caucasian	AA	HB	105/112	−607C/A	PCR-RFLP	0.002
Pawlik et al. [35]	2007	Poland	Caucasian	AA	PB	231/305	−607C/A; −137G/C	Allele-specific PCR	0.75; 0.08
Sebelova et al. [10]	2007	Czech	Caucasian	AR	HB	539/312	−607C/A; −137G/C	PCR-RFLP	0.06; 0.80
Shin et al. [37]	2005	Korea	Asian	AA	PB	438/149	−607C/A; −137G/C	PCR-sequencing	0.14; 0.36
Wu et al. [36]	2012	China	Asian	AA	PB	120/120	−607C/A; −137G/C	PCR-SSP	0.05; 0.40
Chen [39]	2008	China	Asian	AA	HB	82/78	−607C/A; −137G/C	PCR-SSP	0.66; 0.89
Ibrahim et al. [41]	2012	Egypt	Caucasian	AD	HB	25/25	−137G/C	PCR-RFLP	0.10
Trzeciak et al. [42]	2010	Poland	Caucasian	AD	PB	67/46	−137G/C	ARMS-PCR	0.07
Luo et al. [45]	2008	China	Asian	AD	PB	82/100	−607C/A; −137G/C	PCR-SSP	0.62; 0.76
Izakovicova et al. [49]	2010	Czech	Caucasian	Allergic disorder	PB	633/325	−607C/A; −137G/C	Taqman	0.09; 0.71
Lee et al. [46]	2006	China	Asian	AR	HB	160/166	−607C/A	PCR-RFLP	<0.001
Ming et al. [47]	2011	China	Asian	Drug allergy	HB	606/614	−607C/A; −137G/C	PCR-sequencing	0.07; 0.09
Osawa et al. [44]	2007	Japan	Asian	AD	PB	21/100	−607C/A; −137G/C	PCR-sequencing	0.88; 0.48
Kim et al. [48]	2011	Korea	Asian	Drug allergy	PB	275/196	−607C/A; −137G/C	SNaPshot	0.84; 0.17
Kato et al. [43]	2009	Japan	Asian	AD	HB	160/104	−137G/C	PCR-RFLP	0.11
Torres et al. [50]	2010	Spain	Caucasian	Henoch-Schönlein purpura	PB	62/200	−607C/A; −137G/C	Taqman	0.05; 0.10

AA, allergic asthma; AD, allergic dermatitis; AR, allergic rhinitis; PB, population-based controls; HB, hospital-based controls; HWE, Hardy-Weinberg equilibrium; PCR, polymerase chain reaction; RFLP, restriction fragment length polymorphism; SSP, sequence-specific primers.

**Table 2:** Meta-analysis of *IL-18* −607C/A and −137G/C polymorphisms with risk of allergic disease.

	Allele model		Dominant model		Recessive model		Homozygous comparison		Heterozygous comparison	
	OR [95% CI]	*P*	OR [95% CI]	*P*	OR [95% CI]	*P*	OR [95% CI]	*P*	OR [95% CI]	*P*
***IL-18 *−607C/A**	**A allele versus C allele**		**AA + AC versus CC**		**AA versus CC + AC**		**AA versus CC**		**AC versus CC**	
Overall	1.03 [0.91–1.15]	0.67	1.02 [0.85–1.22]	0.86	1.07 [0.88–1.28]	0.51	1.01 [0.77–1.33]	0.94	1.00 [0.83–1.21]	0.98
Type of disease										
AA	0.98 [0.89–1.08]	0.72	0.87 [0.74–1.03]	0.10	1.08 [0.82–1.43]	0.58	0.88 [0.61–1.27]	0.48	0.82 [0.69–0.98]	0.03
AD	1.15 [0.81–1.64]	0.44	1.39 [0.80–2.41]	0.24	1.01 [0.55–1.87]	0.98	1.27 [0.61–2.65]	0.53	1.42 [0.79–2.56]	0.24
AR	1.05 [0.88–1.24]	0.60	1.36 [0.55–3.34]	0.51	1.00 [0.73–1.36]	0.98	1.21 [0.84–1.75]	0.30	1.49 [0.47–4.78]	0.50
Other types	1.02 [0.74–1.43]	0.89	1.02 [0.66–1.60]	0.92	1.09 [0.67–1.78]	0.72	1.09 [0.55–2.18]	0.80	1.03 [0.70–1.51]	0.88
Ethnicity										
Asian	1.04 [0.88–1.24]	0.61	1.10 [0.82–1.46]	0.53	1.02 [0.80–1.31]	0.85	0.96 [0.64–1.45]	0.86	1.12 [0.82–1.52]	0.47
Caucasian	0.97 [0.88–1.08]	0.57	0.90 [0.77–1.04]	0.15	1.14 [0.83–1.56]	0.42	1.02 [0.82–1.27]	0.86	0.86 [0.73–1.01]	0.06
Source of control										
PB	0.93 [0.86–1.01]	0.11	0.86 [0.76–0.98]	0.03	0.97 [0.85–1.11]	0.69	0.80 [0.68–0.95]	0.009	0.85 [0.74–0.98]	0.03
HB	1.27 [1.03–1.57]	0.03	1.36 [0.94–1.96]	0.10	1.46 [0.97–2.21]	0.07	1.75 [1.14–2.68]	0.01	1.27 [0.84–1.94]	0.26

***IL-18 *−137G/C**	**C allele versus G allele**		**CC + CG versus GG**		**CC versus CG + GG**		**CC versus GG**		**CG versus GG**	
Overall	0.93 [0.80–1.07]	0.31	0.97 [0.82–1.14]	0.70	0.77 [0.58–1.02]	0.07	0.76 [0.55–1.04]	0.08	1.02 [0.88–1.18]	0.78
Type of disease										
AA	0.94 [0.86–1.03]	0.21	0.97 [0.87–1.08]	0.63	0.80 [0.63–1.02]	0.07	0.80 [0.63–1.02]	0.07	1.00 [0.90–1.12]	0.95
AD	0.66 [0.30–1.46]	0.30	0.70 [0.29–1.71]	0.44	0.30 [0.15–0.60]	<0.001	0.26 [0.12–0.53]	<0.001	0.84 [0.38–1.82]	0.65
AR	0.93 [0.75–1.16]	0.51	0.94 [0.71–1.25]	0.68	0.81 [0.48–1.37]	0.43	0.80 [0.47–1.37]	0.42	0.97 [0.73–1.30]	0.85
Other types	0.96 [0.61–1.53]	0.87	1.00 [0.60–1.66]	0.99	0.71 [0.47–1.09]	0.12	0.70 [0.46–1.08]	0.11	1.05 [0.66–1.68]	0.84
Ethnicity										
Asian	1.05 [0.85–1.30]	0.64	1.12 [0.89–1.39]	0.34	0.78 [0.54–1.12]	0.18	0.81 [0.56–1.16]	0.25	1.15 [0.93–1.43]	0.20
Caucasian	0.80 [0.66–0.98]	0.03	0.83 [0.68–1.02]	0.08	0.71 [0.49–1.02]	0.06	0.66 [0.43–1.00]	0.05	0.93 [0.83–1.05]	0.24
Source of control										
PB	0.87 [0.73–1.05]	0.14	0.91 [0.76–1.10]	0.35	0.76 [0.51–1.11]	0.15	0.73 [0.48–1.12]	0.15	0.98 [0.87–1.10]	0.72
HB	1.04 [0.77–1.39]	0.81	1.09 [0.79–1.51]	0.60	0.72 [0.54–0.96]	0.03	0.72 [0.53–0.97]	0.03	1.14 [0.85–1.54]	0.38

AA, allergic asthma; AD, allergic dermatitis; AR, allergic rhinitis; PB, population-based control; HB, hospital-based control.
